# Phylogenetic analyses of *Vitis *(Vitaceae) based on complete chloroplast genome sequences: effects of taxon sampling and phylogenetic methods on resolving relationships among rosids

**DOI:** 10.1186/1471-2148-6-32

**Published:** 2006-04-09

**Authors:** Robert K Jansen, Charalambos Kaittanis, Christopher Saski, Seung-Bum Lee, Jeffrey Tomkins, Andrew J Alverson, Henry Daniell

**Affiliations:** 1 Section of Integrative Biology and Institute of Cellular and Molecular Biology, Patterson Laboratories 141, University of Texas, Austin, TX 78712, USA; 2 University of Central Florida, Dept. of Molecular Biology & Microbiology, Biomolecular Science, Building #20, Orlando, FL 32816-2364, USA; 3 Clemson University Genomics Institute, Clemson University, Biosystems Research Complex, 51, New Cherry Street, SC 29634, USA

## Abstract

**Background:**

The Vitaceae (grape) is an economically important family of angiosperms whose phylogenetic placement is currently unresolved. Recent phylogenetic analyses based on one to several genes have suggested several alternative placements of this family, including sister to Caryophyllales, asterids, Saxifragales, Dilleniaceae or to rest of rosids, though support for these different results has been weak. There has been a recent interest in using complete chloroplast genome sequences for resolving phylogenetic relationships among angiosperms. These studies have clarified relationships among several major lineages but they have also emphasized the importance of taxon sampling and the effects of different phylogenetic methods for obtaining accurate phylogenies. We sequenced the complete chloroplast genome of *Vitis vinifera *and used these data to assess relationships among 27 angiosperms, including nine taxa of rosids.

**Results:**

The *Vitis vinifera *chloroplast genome is 160,928 bp in length, including a pair of inverted repeats of 26,358 bp that are separated by small and large single copy regions of 19,065 bp and 89,147 bp, respectively. The gene content and order of *Vitis *is identical to many other unrearranged angiosperm chloroplast genomes, including tobacco. Phylogenetic analyses using maximum parsimony and maximum likelihood were performed on DNA sequences of 61 protein-coding genes for two datasets with 28 or 29 taxa, including eight or nine taxa from four of the seven currently recognized major clades of rosids. Parsimony and likelihood phylogenies of both data sets provide strong support for the placement of Vitaceae as sister to the remaining rosids. However, the position of the Myrtales and support for the monophyly of the eurosid I clade differs between the two data sets and the two methods of analysis. In parsimony analyses, the inclusion of *Gossypium *is necessary to obtain trees that support the monophyly of the eurosid I clade. However, maximum likelihood analyses place *Cucumis *as sister to the Myrtales and therefore do not support the monophyly of the eurosid I clade.

**Conclusion:**

Phylogenies based on DNA sequences from complete chloroplast genome sequences provide strong support for the position of the Vitaceae as the earliest diverging lineage of rosids. Our phylogenetic analyses support recent assertions that inadequate taxon sampling and incorrect model specification for concatenated multi-gene data sets can mislead phylogenetic inferences when using whole chloroplast genomes for phylogeny reconstruction.

## Background

The estimation of phylogenetic relationships among angiosperms has received considerable attention during the past decade with the rapid increase in availability of DNA sequence data from a wide diversity of markers and taxa [reviewed in [1]]. Most previous molecular phylogenetic studies of flowering plants have relied on one to several genes from the chloroplast, mitochondrial, and/or nuclear genomes, though most of these analyses were based on chloroplast markers. These efforts have resolved the relationships among many of the major lineages of angiosperms but a number of outstanding issues remain [1]. Completely sequenced chloroplast genomes provide a rich source of data that can be used to address phylogenetic questions at deep nodes in the angiosperm tree [2-6]. The use of DNA sequences from all of the shared chloroplast genes provides many more characters for phylogeny reconstruction compared to previous studies that have relied on only one or a few genes to address the same questions. However, the whole genome approach can result in misleading estimates of relationships because of limited taxon sampling [5,7-10] and the use of incorrect models of sequence evolution in concatenated datasets [4,11]. Thus, there is a growing interest in expanding the taxon sampling of complete chloroplast genome sequences and developing new evolutionary models for phylogenetic analysis of chloroplast sequences [12] to overcome these concerns.

The rosids represent the largest of the eight major clades of core eudicots and include nearly one third of all flowering plants. Single and multi-gene phylogenies of rosids have identified seven major clades, however, relationships among these clades remain unresolved [13-16]. One of these unresolved clades includes the Vitaceae, which includes grape, an important crop plant. The phylogenetic position of Vitaceae has been controversial for many years. Some previous classifications place the family within the Rhamnales in the subclass Rosidae [17]. More recent molecular phylogenies based on one to four genes provided weak support for the placement of Vitaceae sister to the Caryophylales [18], asterids [18], Saxifragales [14], Dilleniaceae [19], or to the rosids [14-16]. Thus, the phylogenetic relationship of the grape family to core eudicots remains unresolved.

In this article, we report on the complete sequence of the chloroplast genome of grape (*Vitis vinifera*, Vitaceae). In addition to describing the organization of the chloroplast genome, we present results of phylogenetic analyses of DNA sequences for 61 genes from grape and 26 other angiosperm chloroplast genomes, including eight other members of the rosid clade. The phylogenetic analyses provide insights into the relationship of Vitaceae to other rosids and illustrate the importance of taxon sampling and analytical method on addressing phylogenetic questions using whole genome sequences. The complete chloroplast genome sequence of *Vitis *also provides valuable data for using chloroplast genetic engineering for this economically important crop plant [20].

## Results

### Size, gene content, order and organization of the grape chloroplast genome

**Figure 1:**
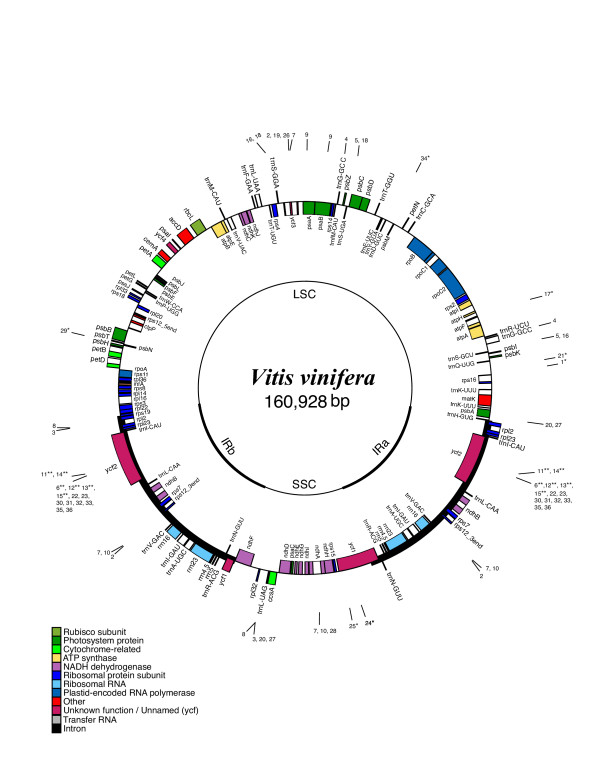
Gene map of the *Vitis vinifera *chloroplast genome. The thick lines indicate the extent of the inverted repeats (IRa and IRb), which separate the genome into small (SSC) and large (LSC) single copy regions. Genes on the outside of the map are transcribed in the clockwise direction and genes on the inside of the map are transcribed in the counterclockwise direction. Numbers on the outside of map indicate location of repeats in Table 1. Repeats indicated by * (palindrome) and ** (tandem) are only shown once since they occur in the same location.

The complete chloroplast genome of grape is 160,928 bp in length (Fig. 1) and includes a pair of inverted repeats 26,358 bp long, separated by a small and a large single copy region of 19,065 bp and 89,147 bp, respectively. The grape chloroplast genome has 113 unique genes, 18 of which are duplicated in the IR, for a total of 131 genes (Fig. 1). There are four ribosomal and 30 distinct tRNA genes; seven of the tRNA genes and all rRNA genes are duplicated within the IR. There are 17 intron-containing genes, 15 of which contain one intron, and two of which contain two introns. Overall, the gene order in the grape chloroplast genome is identical to that of tobacco. The grape genome is 37.40% GC and 62.60% AT; 57.55% of the genome corresponds to coding regions and 42.45% to non-coding regions, including introns and intergenic spacers.

### Repeat structure

**Figure 2:**
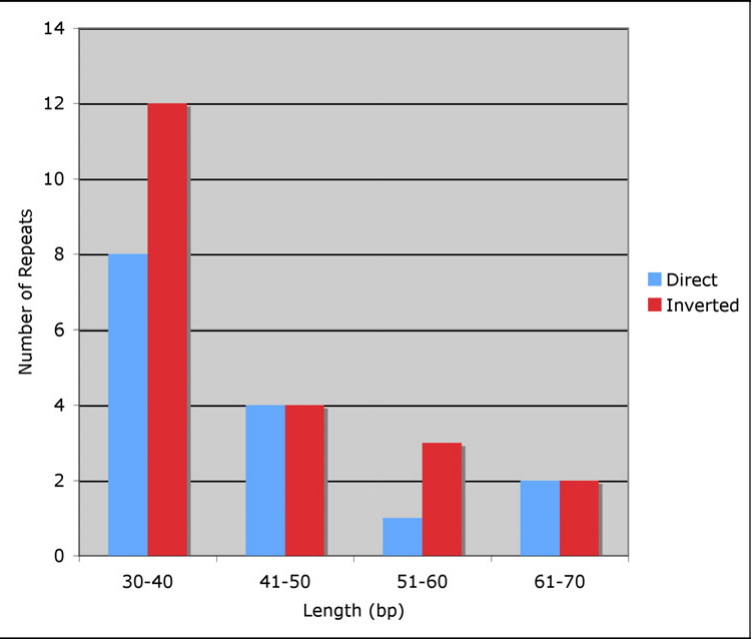
Histogram showing the number of repeated sequences ≥ 30 bp long with a sequence identity ≥ 90% in the grape chloroplast genome.

Repeat analysis identified 36 repetitive elements (30 bp or longer with a sequence identity of at least 90%), 15 of which are direct repeats and 21 of which are inverted repeats (Fig. 2 and Table 1). Eight direct repeats and 12 inverted repeats were 30 – 40 bp long, and the longest direct repeats were 64 bp. The majority of the repeats were located within intergenic spacer regions, intron sequences and *ycf2*. Two distinct 64 bp direct repeats were found in *ycf2*, which is located in the IR. Additionally, a 41 bp direct repeat was located in *psaA *and *psaB*, and a shorter, 32 bp direct repeat was found in two serine transfer-RNA (*trnS*) genes that recognize different codons; *trnS-GCU *and *trnS-UGA*. Lastly, a 31-bp direct repeat was identified within *trnG-GCC *in the IR, and a 39-bp direct repeat was found three times in the grape chloroplast genome, with a single occurrence in an intergenic spacer region, and also in the *ycf3 *and *ndhA *introns.

**Table 1:** Location of repeats in the grape chloroplast genome. Repeats 1 to 15 are direct, and 16 to 36 are inverted. Table includes repeats at least 30 bp in size, with a sequence identity ≥ 90%. IGS = Intergenic spacer. See Figure 1 for location of repeats on the gene map. Repeats indicated by * (palindrome) and ** (tandem) are only shown once on the circular map in Figure 1.

**Repeat Number**	**Size (bp)**	**Location**
1	30*	IGS
2	30	*ycf3 *intron, IGS
3	31	IGS
4	31	*TrnG-GCC*
5	32	IGS (4 bp) – *trnS-GCU*, IGS (4 bp) – *trnS-UGA*
6	34**	*ycf2*
7	39	*ycf3 *intron, IGS, *ndhA *intron
8	40	IGS
9	41	*psaB *exon – *psaA *exon
10	42	IGS, *ndhA *intron
11	46**	*ycf2*
12	46**	*ycf2*
13	52**	*ycf2*
14	64**	*ycf2*
15	64**	*ycf2*
16	30	IGS (3 bp) – *trnS-GCU*, *trnS-GGA*
17	30*	IGS
18	30	IGS (2 bp) – *trnS-UGA*, *trnS-GGA*
19	30	*ycf3 *intron, IGS
20	31	IGS
21	33*	IGS
22	34	*ycf2*
23	34	*ycf2*
24	34*	*ycf1*
25	36*	IGS, *ycf1*
26	39	*ycf3*, IGS
27	40	IGS
28	42	*ndhA *intron, IGS
29	43*	IGS
30	46	*Ycf2*
31	46	*Ycf2*
32	52	*Ycf2*
33	52	*Ycf2*
34	54*	IGS
35	64	*Ycf2*
36	64	*Ycf2*

### RNA variable sites in grape chloroplast transcripts

Comparison of DNA and EST sequences for chloroplast-encoded proteins retrieved from GenBank showed that most photosynthetic machinery and ribosomal subunit genes have 100% sequence identity with their respective EST sequences. Eleven non-synonymous nucleotide substitutions, resulting in a total of nine amino acid changes, were identified for *atpI*, *clpP*, *matK*, *petB*, *petD*, *psbA *and *rpl22 *compared to the ESTs (Table 2). Also, there were five synonymous substitutions. In the cases of non-synonymous substitutions, all genes experienced one nucleotide substitution except *clpP*, which had five variable sites. Lastly, in *atpI*, *clpP *and *psbA *the nucleotide substitutions had an impact on the hydropathy of the amino acid, changing it from aliphatic to hydrophilic, and vice versa. These differences could be due to mRNA editing, sequencing error of either the genomic DNA or ESTs, or polymorphisms between the samples used for genomic and EST sequences (see Discussion).

**Table 2:** Differences observed by comparison of grape chloroplast genome sequences with EST sequences obtained by BLAST search in Genbank.

**Gene**	**Gene size (bp)**	**EST Sequence**^**a**^	**Number of variable sites**	**Variation type**^**b**^	**Position(s)**^**c**^	**Amino acid change**
*atpI*	744	1–744	2	G-A C-U	453 629	A-A L-S
						
*clpP*	591	62–366	5	G-A A-U	64 65	D-I
				T-A A-U	70 71	Y-I
				C-U	364	R-W
						
*matK*	1509	416–1262	2	C-U G-A	448 1260	H-Y K-K
						
*ndhA*	1092	1–553	1	T-C	553	L-L
						
*ndhI*	504	77–356	1	C-U	162	R-R
						
*PetB*	648	4–648	1	G-A	5	S-N
						
*PetD*	483	6–483	1	G-A	7	V-I
						
*PsbA*	1062	397–1014	2	T-C G-A	420 463	R-R A-T
						
*rpl22*	462	1–462	1	C-U	46	Q-Stop

^a^Sequence analyzed coordinates based on the gene sequence, considering the first base of the initiation codon as bp 1. ^b^Variation type: (nucleotide in genomic DNA) – (nucleotide in mRNA). ^c^Variable position is given in reference to the first base of the initiation codon of the gene sequence.

### Phylogenetic analysis

We examined two datasets that differed by a single rosid taxon to assess the effect of taxon sampling on resolving relationships among rosids. The first data matrix examined for phylogenetic analyses included 61 protein-coding genes for 28 taxa (Table 3, excluding *Gossypium*), including 26 angiosperms and two gymnosperm outgroups (*Pinus *and *Ginkgo*), and the second data matrix included 29 taxa with the addition of *Gossypium*. Both data sets comprised 45,573 nucleotide positions but when the gaps were excluded there were 39,624 characters.

**Table 3:** Taxa included in phylogenetic analyses with GenBank accession numbers and references.

Taxon	GenBank Accession Numbers	Reference
Gymnosperms – Outgroups		
*Pinus thunbergii*	NC_001631	Wakasugi et al. 1994 [84]
*Ginkgo biloba*	DQ069337-DQ069702	Leebens-Mack et al 2005 [5]
Basal Angiosperms		
*Amborella trichopoda*	NC_005086	Goremykin et al. 2003 [3]
*Nuphar advena*	DQ069337-DQ069702	Leebens-Mack et al 2005 [5]
*Nymphaea alba*	NC_006050	Goremykin et al. 2004 [2]
Monocots		
*Acorus americanus*	DQ069337-DQ069702	Leebens-Mack et al 2005 [5]
*Oryza sativa*	NC_001320	Hiratsuka et al. 1989 [85]
*Saccharum officinarum*	NC_006084	Asano et al. 2004 [86]
*Triticum aestivum*	NC_002762	Ikeo and Ogihara, unpublished
*Typha latifolia*	DQ069337-DQ069702	Leebens-Mack et al 2005 [5]
*Yucca schidigera*	DQ069337-DQ069702	Leebens-Mack et al 2005 [5]
*Zea mays*	NC_001666	Maier et al. 1995 [87]
Magnoliids		
*Calycanthus floridus*	NC_004993	Goremykin et al. 2003 [43]
Eudicots		
*Arabidopsis thaliana*	NC_000932	Sato et al. 1999 [88]
*Atropa belladonna*	NC_004561	Schmitz-Linneweber et al. 2002 [57]
*Cucumis sativus*	NC_007144	Plader et al. unpublished
*Eucalyptus globulus*	AY780259	Steane 2005 [89]
*Glycine max*	DQ317523	Saski et al. 2005 [49]
*Gossypium hirsutum*	DQ345959	Lee et al. [55]
*Lotus corniculatus*	NC_002694	Kato et al. 2000 [42]
*Medicago truncatula*	NC_003119	Lin et al., unpublished
*Nicotiana tabacum*	NC_001879	Shinozaki et al. 1986 [90]
*Oenothera elata*	NC_002693	Hupfer et al. 2000 [44]
*Panax schinseng*	NC_006290	Kim and Lee 2004 [91]
*Ranunculus macranthus*	DQ069337-DQ069702	Leebens-Mack et al 2005 [5]
*Solanum lycopersicum*	DQ347959	Daniell et al. [92]
*Solanum bulbocastanum*	DQ347958	Daniell et al. [92]
*Spinacia oleracea*	NC_002202	Schmitz-Linneweber et al. 2001 [93]
*Vitis vinifera*	DQ424856	Current study

**Figure 3:**
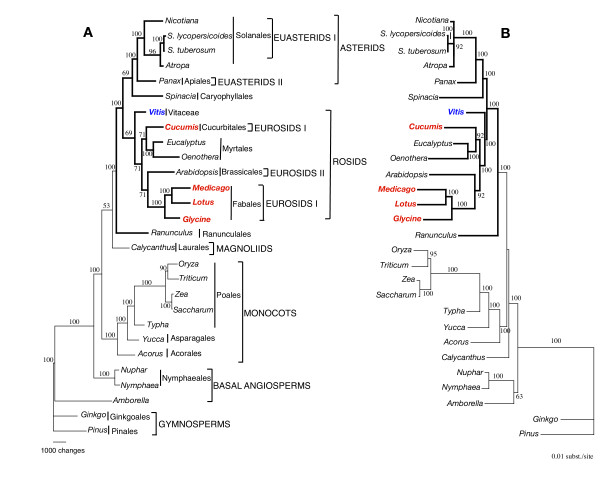
Phylogenetic tree of 28-taxon data set based on 61 chloroplast protein-coding genes using maximum parsimony (MP) and maximum likelihood (ML). (A) The MP tree has a length of 49,511, a consistency index of 0.47 (excluding uninformative characters) and a retention index of 0.62. (B) The ML tree has a ML value of – lnL = 289638.676. Numbers above and below nodes are bootstrap support values ≥ 50%. Ordinal and higher level group names follow APG II [94]. Taxa in red are members of eurosid I and *Vitis *is indicated in blue. Thicker lines in tree indicate members of Eudicots.

**Figure 4:**
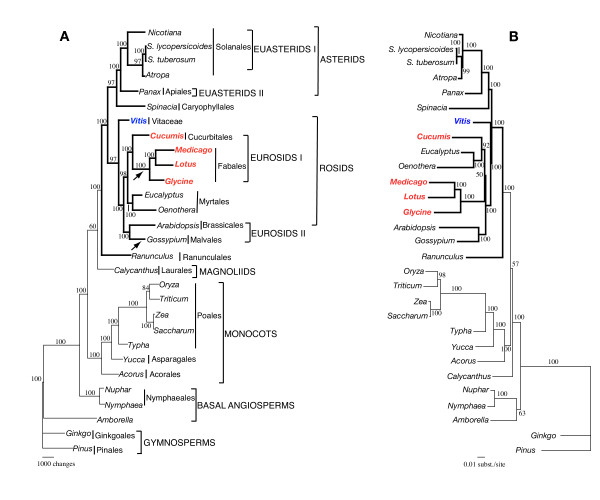
Phylogenetic trees of 29-taxon data set (including *Gossypium*) based on 61 chloroplast protein-coding genes using maximum parsimony (MP) and maximum likelihood (MP). (A) The MP tree has a length of 51,056, a consistency index of 0.46 (excluding uninformative characters) and a retention index of 0.61. (B) The ML tree has a ML value of – lnL = 296670.545. Numbers at nodes indicate bootstrap support ≥ 50%. Arrows indicate taxa that have lost the *rpl22 *gene. Ordinal and higher level group names follow APG II [94]. Taxa in red are members of eurosid I and *Vitis *is indicated in blue. Thicker lines in tree indicate members of Eudicots.

Maximum Parsimony (MP) analyses of the 28-taxon dataset resulted in a single, fully resolved tree with a length of 49,511, a consistency index of 0.47 (excluding uninformative characters) and a retention index of 0.62 (Fig. 3A). Bootstrap analyses indicated that 18 of the 25 nodes were supported by values ≥ 95% and all but one of these had a bootstrap value of 100%. Maximum likelihood (ML) analysis resulted in a single tree with – lnL = 289638.676. ML bootstrap values also were consistently high, with values of ≥ 95% for 21 of the 25 nodes. The ML and MP trees had very similar topologies, except for two important differences. The first concerned the position of the two basal angiosperm lineages. The MP tree placed *Amborella *as the most basal lineage followed by the Nymphaeales (including *Nuphar *and *Nymphaea*), whereas the ML tree placed *Amborella *sister to the Nymphaeales, and together this group formed the basal lineage of angiosperms. The second topological difference concerned the placement of *Calycanthus*, the only representative of the magnolids. The MP tree placed *Calycanthus *sister to the eudicots, whereas the ML tree positioned *Calycanthus *as sister to a large clade that included both monocots and eudicots. Support for the different placements of *Calycanthus *was weak in both MP and ML analyses, whereas the support for the different resolutions of basal angiosperms was stronger (Fig. 3). These two differences were also detected in a recent phylogeny of basal angiosperms based on whole chloroplast genome sequences [5]. The remaining angiosperms formed two major clades, one including monocots and a second including the eudicots (highlighted with thick lines in Figs. 3 and 4). Monophyly of the monocots was strongly supported (100% bootstrap value for both MP and ML) and included members of three different orders (Acorales, Asparagales, and Poales). Ranunculales were the earliest diverging lineage of eudicots. There were two major clades of core eudicots, one including the rosids and the second including the Caryophyllales + asterids. Within the rosids, *Vitis *was sister to the remaining taxa, which formed two clades, one including *Cucumis *(Cucurbitaceae) + Myrtales, and a second with *Arabidopsis *(Brassicales) + Fabales. Overall, relationships within rosids were in agreement with recent phylogenies [summarized in [1]] except that the eurosids I clade was paraphyletic in our analyses.

MP analysis of the second dataset of 29 taxa including *Gossypium *resulted in a single most parsimonious tree with a length of 51,056, a consistency index of 0.46 (excluding uninformative characters) and a retention index of 0.61 (Fig. 4A). Bootstrap analyses indicated that 24 of the 26 nodes were supported by values ≥ 95%, and all but four of these nodes had a bootstrap value of 100%. ML analysis resulted in a tree with a – lnL = 296670.545 (Fig. 4B). ML bootstrap analyses indicated that 22 of the 26 nodes were supported by values ≥ 95% and all but two of these nodes had a bootstrap value of 100%. Both MP and ML analyses provided strong support for *Vitis *as the earliest diverging lineage of rosids, monophyly of Myrtales, and sister relationship of Brassicales and Malvales. The ML and MP trees had three important topological differences. The first two differences concerned the position of *Calycanthus *and the basal angiosperms, which were identical to those described above for the analyses that excluded *Gossypium*. The other difference concerned relationships among rosids. The MP tree (Fig. 4A) showed strong support (100% bootstrap) for monophyly of the eurosid I clade because of the sister relationship between the Fabales and Cucurbitales. In contrast, the ML tree indicated that eurosids I are paraphyletic because Cucurbitales were sister to Mrytales rather than Fabales (Fig. 4B); bootstrap support for this relationship was also strong (92%).

## Discussion

Grapes are an important crop plant grown for wine, juice, raisins, and as fresh fruit. In 2004, the world's grape harvest area in 89 grape-producing countries was 7.5 million hectares, and in the United States grapes were grown in 380,000 hectares [21]. The total production of grapes in the US in 2004 was 5,418,160 metric tons and this generated $2.5 billion [21]. There is considerable interest in using chloroplast genetic engineering as an environmentally friendly approach for engineering disease resistance to powdery and downy mildew, two fungal diseases that have a negative impact on the grape industry. Chloroplast genetic engineering offers a number of unique advantages, including a high-level of transgene expression [23], multi-gene engineering in a single transformation event [23-26], transgene containment via maternal inheritance [27-29] or cytoplasmic male sterility [30], and lack of gene silencing, position effect, pleiotropic effects, and undesirable foreign DNA [20,31-35]. Thus far, transgenes have been stably integrated and expressed via the chloroplast genome to confer several useful agronomic traits, including insect resistance [36,37,23], herbicide resistance [27,38], disease resistance [39], drought tolerance [31], salt tolerance [40], and phytoremediation [24]. The complete grape chloroplast genome sequence reported in this paper provides valuable characterization of spacer regions for potential integration of transgenes at optimal sites via homologous recombination, as well as endogenous regulatory sequences for optimal expression of transgenes.

### Genome organization and evolution

The organization of the *Vitis *genome with two copies of an IR separating the SSC and LSC regions is identical to most sequenced angiosperm chloroplast genomes [reviewed in [41]]. The size of the genome at 160,928 bp is also within the known size range for angiosperms, which generally vary from 150,519 (*Lotus *[42]) to 162,686 bp (*Amborella *[3]) among photosynthetic genomes from dicots that have both copies of the IR. Size of the *Vitis *IR at 26,358 bp is also well within the size range of other sequenced dicot genomes, which range from 23,302 (*Calycanthus *[43]) to 27,807 bp (*Oenothera *[44]). Gene content and order of the *Vitis *chloroplast genome is virtually identical to tobacco and many other unrearranged angiosperm chloroplast genomes. Several previously sequenced rosid chloroplast genomes have lost the *rpl22 *gene, including legumes [45-49]. The distribution of this loss on the chloroplast phylogeny (arrows in Fig. 4A) indicates that there have been at least two independent losses of *rpl22 *in rosids. Multiple, independent gene losses in angiosperms have been demonstrated for other genes including *infA *[50], *rps16 *[48] and *accD *[51,52]. Thus, it is evident that gene losses are not always reliable indicators of phylogenetic relationships.

It is increasingly evident that chloroplast genomes contain repeated sequences other than the IR [49]. Several studies have identified a higher incidence of dispersed repeats in genomes that have experienced extensive rearrangements [53,54]. However, dispersed repeats are also being detected in unrearranged genomes. In most cases, these repeats are more common in intergenic spacers and introns, which is also true for the *Vitis *genome. Repeats have been located in a number of other rosids [49] in the same regions as those identified in the *Vitis *genome. One of these, a 32 bp repeat in the *trnS *gene, is in the same location in *Gossypium hirsutum *[55], indicating that this repeat may be shared among rosids. Although repeats have been implicated in playing a role in chloroplast genome rearrangements [56], their effect if any in unrearranged chloroplast genomes is unknown.

Based on previous studies of *Atropa *[57] and tobacco [58], posttranscriptional RNA editing events, as well as deamination-facilitating attacks on nucleotides' exocyclic amino groups, yield primarily C-to-U alterations. Analyses of the *Vitis *chloroplast genome and the corresponding ESTs indicate that the five C-to-U changes likely represent mRNA edits. However, the remaining six differences could be either sequencing errors in the genomic DNA or EST sequences or due to the use of different cultivars and/or plants/tissues used for sequencing. Our methods eliminate the latter explanation since we only compared DNA and EST sequences from leaves of the chardonnay variety of *Vitis vinifera*. In view of the high depth of coverage (8X) of our genomic DNA sequences, we believe that the non C-to-U changes represent EST sequencing errors.

Evolutionary loss of RNA editing sites has been observed in earlier studies and could be attributed to a decrease in the effect of RNA-editing enzymes [59]. Additionally, conversions other than C-to-U in *Vitis *and other plants suggest that chloroplast genomes may be accumulating a considerable number of nucleotide substitutions, and some genes might accumulate more changes than others, such as the *petL *and *ndh *genes that have a high frequency of RNA editing [60]. Therefore, despite high levels sequence conservation in chloroplast genomes, variations do occur posttranscriptionally, promoting translational efficiency due to transcript-protein complex binding and/or changes in the chloroplasts microenvironment (e.g., like redox potential or light intensity [61,62]).

### Phylogenetic implications

Phylogenetic analyses of 28 (Fig. 3) or 29 (Fig. 4) angiosperms based on 61 protein-coding genes identified many of the major lineages recognized in previous phylogenetic hypotheses of flowering plants [reviewed in [1]]. Two groups, *Amborella *and Nymphaelaes (represented by *Nuphar *and *Nymphaea*) are basal, with *Amborella *forming the first diverging lineage in MP analyses and *Amborella*/Nymphaelaes together forming the most basal clade in ML trees. These results are congruent with recent 61-gene analyses by Leebens-Mack et al. [5] and support their contention that limited taxon sampling in earlier whole chloroplast genome phylogenies led some previous workers to suggest that *Amborella *may not be among the earliest diverging angiosperm lineages [2,3]. Monophyly of the monocots is strongly supported, and they are sister to the remaining angiosperms. *Calycanthus*, the sole representative of the magnolids, is weakly supported as sister to eudicots in the MP analyses (Figs. 3A and 4A) but the genus is weakly supported as sister to a clade that includes both monocots and eudicots in ML trees (Figs. 3B and 4B). Monophyly of eudicots is strongly supported (100% bootstrap values), in agreement with phylogenies based on both pollen [63,64] and other molecular data [13,14,18,19,65-67]. Within eudicots, Ranunculales diverge first and are sister to a strongly supported eudicot clade that includes two moderately to well-supported groups comprising the rosids and asterids. The early divergence of Ranunculales among eudicots is in agreement with many recent molecular phylogenies [see chapter 5 in [1]]. Although previous studies have clearly indicated that Carylophyllales belong in the core eudicot clade [1], resolution of the relationships of Caryophyllales to other major clades of eudicots remains uncertain. This order has been considered to be closely allied to rosids, asterids, or simply as an unresolved major eudicot clade sister to the Dilleniaceae [15]. Although taxon sampling is limited in our 61 gene phylogeny, there is moderate to strong support for a sister relationship between the Caryophyllales and asterids (Figs. 3 and 4).

The rosid clade is very diverse, including nearly 140 families representing approximately 39% of the species of angiosperms. The most recent phylogenies of this group [summarized in chapter 8 in [1]] indicate that there are seven major clades whose relationships still remain unresolved. Eight (Fig. 3) or nine (Fig. 4, includes *Gossypium*) representatives of four of these major clades are included in our phylogenetic analyses, including members of eurosids I, eurosids II, Myrtales, and Vitaceae. Phylogenetic analyses of both datasets using MP and ML clearly indicate that the Vitaceae is sister to the remaining rosids, and therefore represents an early diverging member of the rosid clade. Previous molecular phylogenetic comparisons that included Vitaceae could not resolve its relationship. Phylogenetic analyses of *rbcL *sequences alone placed the Vitaceae as sister to either the Caryophyllales or asterid clade with weak support [18]. Phylogenies based on *atpB *provided only weak support for a sister relationship of Vitaceae to Saxifragales [14]. Several phylogenies based on two to four genes suggested that the Vitaceae are sister to rest of the rosids, with relatively weak support (50–75%; [14-16]). However, phylogenies based on the chloroplast gene *matK *did not place Vitaceae sister to rosids but instead positioned the family as sister to Dilleniaceae with weak support [19]. In short, the phylogenetic position of Vitaceae is equivocal, though our results strongly support the earlier findings that Vitaceae represent an early diverging clade within rosids (Figs. 3 and 4).

The two datasets we examined differed by only one taxon but the results of MP analyses differed dramatically regarding the placement of three of the four rosid clades examined (compare Figs. 3A and 4A). The 28-taxon dataset (excluding *Gossypium*) showed relationships that are incongruent with recent molecular phylogenies of rosids [1] by placing the eurosids II (represented by only Brassicales) sister to the Fabales in eurosids I. This made eurosids I paraphyletic because the other representative of this clade is *Cucumis *(Cucurbitales), which is sister to the Brassicales in molecular phylogenies of rosids [1]. The addition of *Gossypium *in the 29-taxon dataset generates MP trees (Fig. 4A) that are congruent with previous angiosperm phylogenies. The Brassicales and Malvales are sister and there is strong support for the monophyly of eurosid II. The addition of *Gossypium *also makes the eurosid I clade strongly monophyletic in the MP tree by placing the Cucurbitales sister to the Fabales, both of which are members of the nitrogen-fixing clade [see chapter 8 in [1]]. In contrast to the MP trees, relationships among the major rosid clades do not differ in the ML trees when *Gossypium *is added. In both the 28 and 29-taxon data sets the ML trees do not support the monophyly of eurosids I since Cucurbitales (eurosid I) are sister to the Myrtales and Brassicales (eurosid II) are sister to the Fabales (eurosid I). Therefore, the ML analyses are incongruent with currently accepted relationships among rosids [1], though the strongest support for the monophyly of eurosid I clade is only 77% (jackknife support) in a three-gene analysis [15]. Thus, our results suggest that additional phylogenetic studies are needed to assess the monophyly of eurosids I and their relationship to other rosids.

There has been considerable debate regarding the utility of whole genome sequences for phylogeny reconstruction [5,7-10]. Some have argued that the use of more genes from whole genomes has great potential for providing much more data for resolving phylogenetic relationships [2,68], whereas others have suggested that problems with limited taxon sampling available for whole genomes [5,7,8,10] and model misspecification [4,11] overshadows any potential advantages. One example that highlighted each of these concerns centered around the controversy regarding identification of basal angiosperms. Leebens-Mack et al. [5] demonstrated that inadequate taxon sampling clearly played a role in misleading some previous studies, and Goremykin et al. [4] demonstrated that ML analyses of whole chloroplast genome data sets can be sensitive to model specification. It is well known that ML methods fail when model parameters are misspecified [69-71]. The phylogenetic analyses in this study provide yet another example of these phenomena. Addition of the *Gossypium *genome to our parsimony analyses generated trees that are congruent with current understanding of relationships among the major rosid clades. However, the ML analyses are incongruent with the MP trees regarding the monophyly and relationships of the rosid clades and support for the alternative relationships was very strong in each case (compare Figs. 4A and 4B). It is possible, if not likely, that the use of a single "average" model (GTR + I + Γ) in the ML analyses is inappropriate for a data set of 61 concatenated genes [see [11] for a discussion of this issue]. Future phylogenetic analyses of complete chloroplast genome sequences should consider using methods in which different models can be applied to different partitions of the data (e.g., genes, codon positions, functional groups) [72]. Development of more appropriate models of evolution of chloroplast sequences [12] may also improve the accuracy of phylogenies based on these genomes. Thus, we need more extensive sampling of whole chloroplast genomes from the major lineages of flowering plants and more rigorous phylogenetic analyses before the full potential of this approach can be realized. Ongoing projects by several labs [see [73] for a list of some of these] should greatly enhance our taxon sampling so that we can generate reliable phylogenies based on whole chloroplast genomes.

## Conclusion

The chloroplast genome of *Vitis *has a very similar size and organization to other previously sequenced, unrearranged angiosperm chloroplast genomes. These sequences will provide a valuable resource for developing transgenes for this important crop plant using the more environmentally friendly chloroplast genetic engineering technology [20]. Phylogenetic analyses of a dataset of sequences of 61 shared protein-coding genes of *Vitis *and 26 other angiosperm genomes demonstrated the importance of taxon sampling and methods of phylogenetic analysis for phylogenomic studies. Furthermore, trees generated by both parsimony and likelihood methods provided support for the resolution of relationships among eudicots. This included support for the position of the Ranunculales as the earliest diverging lineage of eudicots, the placement of the Caryophyllales as the sister clade to the asterids, and the position of the Vitaceae sister to all other rosids. However, resolution of relationships among the remaining rosid clades based on complete chloroplast genome sequences remains unresolved due to limited taxon sampling and differences in trees generated by MP and ML analyses.

## Methods

### DNA sources

The bacterial artificial chromosome (BAC) library of grape was constructed by ligating size-fractionated partial *Hind *III digests of total cellular, high molecular weight DNA with the pINDIGOBAC vector. The average insert size of the grape library is 144 kb. BAC-related resources for this public library can be obtained online from the Clemson University Genomics Institute BAC/EST Resource Center [74].

BAC clones containing the chloroplast genome inserts were isolated by screening the library with a soybean chloroplast probe. The first 96 positive clones from screening were pulled from the library, arrayed in a 96-well microtitre plate, copied, and archived. Selected clones were then subjected to *Hind *III fingerprinting and *Not *I digests. End-sequences were determined and localized on the chloroplast genome of *Arabidopsis thaliana *to deduce the relative positions of the clones, then clones that covered the entire chloroplast genome of grape were chosen for sequencing.

### DNA sequencing and genome assembly

The nucleotide sequences of the BAC clones were determined by the bridging shotgun method. The purified BAC DNA was subjected to hydroshearing, end repair, and then size-fractionated by agarose gel electrophoresis. Fractions of approximately 3.0–5.0 kb were eluted and ligated into the vector pBLUESCRIPT IIKS+. The libraries were plated and arrayed into 40 96-well microtitre plates for the sequencing reactions.

Sequencing was performed using the Dye-terminator cycle sequencing kit (Perkin Elmer Applied Biosystems, USA). Sequence data from the forward and reverse priming sites of the shotgun clones were accumulated. Sequence data equivalent to eight times the size of the genome was assembled using Phred/Phrap programs [75].

### Gene annotation

The *Vitis *genome was annotated using DOGMA (Dual Organellar GenoMe Annotator) [76], after uploading a FASTA-formatted file of the complete plastid genome to the program's server. BLASTX and BLASTN searches against a custom database of previously published plastid genomes identified *Vitis*' putative protein-coding genes, and tRNAs or rRNAs. For genes with low sequence identity, manual annotation was performed, after identifying the position of the start and stop codons, as well as the translated amino acid sequence, using the plastid/bacterial genetic code.

### Examination of repeat structure

REPuter [77] was used in order to locate and count the direct (forward) and inverted (palindromic) repeats within the grape chloroplast genome. For repeat identification, the following constraints were set to REPuter: (i) minimum repeat size of 30 bp, and (ii) 90% or greater sequence identity, based on Hamming distance of 3 [49]. Manual verification of the identified repeats was performed in EditSeq, while performing intragenomic blast search of the identified repeat sequence.

### Variation between coding sequences and cDNAs

Each of the gene sequences from the grape chloroplast genome was used to perform a BLAST search of expressed sequence tags (ESTs) from Genbank. In order to incorporate specificity into our analyses, the matching ESTs had to meet all of the following criteria: (1) belong to a *Vitis vinifera *cv., (2) belong to the chardonnay variety, and (3) come from leaf tissue. Due to length variations between the screened ESTs and the related gene, the retrieved EST with the highest bit score was selected for further analyses. The retrieved *Vitis vinifera *EST was aligned with the corresponding annotated gene using ClustalX [78], followed by screening for nucleotide and amino acid changes using Megalign and the plastid/bacterial genetic code. Because of variations in the length between an EST and the related gene, the length of the analyzed sequence was recorded.

### Phylogenetic analysis

The 61 genes included in the analyses of Goremykin et al. [2,3] and Leebens-Mack et al. [5] were extracted from our new chloroplast genome sequences of *Vitis *using the organellar genome annotation program DOGMA [76]. The same set of 61 genes was extracted from chloroplast genome sequences of six other recently sequenced angiosperm chloroplast genomes, including tomato, potato, soybean, cotton, cucumber, and *Eucalyptus *(see Table 3 for complete list of genomes examined). In general, alignment of the DNA sequences was straightforward and simply involved adding the 61 genes for the new angiosperms to the aligned data matrix from Leebens-Mack et al. [5]. In some cases, small in-frame insertions or deletions were required for correct alignment. For two genes, *ccsA *and *matK*, the DNA sequences were more divergent, requiring alignment using ClustalX [78] followed by manual adjustments. The complete nucleotide alignment is available online at [79].

Phylogenetic analyses using maximum parsimony (MP) and maximum likelihood (ML) were performed using PAUP* version 4.10 [80] on two data sets, one including 28 taxa and a second including 29 taxa by the addition of *Gossypium*. Phylogenetic analyses excluded gap regions. All MP searches included 100 random addition replicates and TBR branch swapping with the Multrees option. Modeltest 3.7 [81] was used to determine the most appropriate model of DNA sequence evolution for the combined 61-gene dataset. Hierarchical likelihood ratio tests and the Akaikle information criterion were used to assess which of the 56 models best fit the data, which was determined to be GTR + I + Γ by both criteria. For ML analyses we performed an initial parsimony search with 100 random addition sequence replicates and TBR branch swapping, which resulted in a single tree. Model parameters were optimized onto the parsimony tree. We fixed these parameters and performed a ML analysis with three random addition sequence replicates and TBR branch swapping. The resulting ML tree was used to re-optimize model parameters, which then were fixed for another ML search with three random addition sequence replicates and TBR branch swapping. This successive approximation procedure was repeated until the same tree topology and model parameters were recovered in multiple, consecutive iterations. This tree was accepted as the final ML tree (Figs. 3B, 4B). Successive approximation has been shown to perform as well as full-optimization analyses for a number of empirical and simulated datasets [82]. Non-parametric bootstrap analyses [83] were performed for MP analyses with 1000 replicates with TBR branch swapping, 1 random addition replicate, and the Multrees option and for ML analyses with 100 replicates with NNI branch swapping, 1 random addition replicate, and the Multrees option.

## Abbreviations

IR inverted repeat; SSC, small single copy; LSC, large single copy, bp, base pair; ycf, hypothetical chloroplast reading frame; rrn, ribosomal RNA; MP, maximum parsimony; ML, maximum likelihood, EST, expressed sequence tags; cDNA, complementary DNA.

## Authors' contributions

RKJ assisted with extracting and aligning DNA sequences, assisted in phylogenetic analyses, and wrote several sections of this manuscript; CK performed the repeat analyses, comparisons of DNA and EST sequences, assisted with extraction and alignment of DNA sequences for phylogenetic analyses; SBL performed genome annotation; CS & JT performed DNA sequencing and genome assembly; AJA performed phylogenetic analyses; HD conceived and designed this study, interpreted data, wrote several sections and revised several versions of this manuscript. All authors have read and approved the final manuscript.
